# “Naloxone? Not for me!” First cross-assessment by patients and healthcare professionals of the risk of opioid overdose

**DOI:** 10.1186/s12954-024-00941-y

**Published:** 2024-01-23

**Authors:** Aurélie Aquizerate, Morgane Rousselet, Axel Cochard, Marylène Guerlais, Marie Gerardin, Emilie Lefebvre, Mélanie Duval, Edouard-Jules Laforgue, Caroline Victorri-Vigneau

**Affiliations:** 1 Nantes Université, CHU Nantes, Centre d’Evaluation et d’Information sur la Pharmacodépendance-Addictovigilance (CEIP-A), Service de Pharmacologie Clinique, 44000 Nantes, France; 2 Nantes Université, Univ Tours, CHU Nantes, CHU Tours, INSERM, MethodS in Patients-Centered Outcomes and HEalth Research, SPHERE, 44000 Nantes, France

**Keywords:** Naloxone, Opioid overdose, Cross-assessment, Opioid substitution treatment

## Abstract

**Background:**

Opioid-related mortality is a rising public health concern in France, where opioids were in 2021 implicated in 75% of overdose deaths. Opioid substitution treatment (OST) was implicated in almost half of deaths related to substance and drug abuse. Although naloxone could prevent 80% of these deaths, there are a number of barriers to the distribution of take-home naloxone (THN) among opioid users in France. This study is the first one which compares patients' self-assessment of the risk of future opioid overdose with the hetero-assessment provided by healthcare professionals in a population of individuals eligible for naloxone.

**Methods:**

This was a multicenter descriptive observational study carried out in pharmacies across the Pays de la Loire region (France) during April and May 2022. All adult patients who visited a participating pharmacy for a prescription of OST and provided oral informed consent were enrolled in the study. Retrospective data were collected through cross-sectional interviews conducted by the pharmacist with the patient, utilizing an ad hoc questionnaire. The patient’s self-assessment of overdose risk was evaluated using a Likert scale from 0 to 10. The pharmacist relied on the presence or absence of overdose risk situations defined by the French Health Authority (HAS). The need to hold THN was assessed using a composite criterion.

**Results:**

A total of 34 patients were interviewed; near one third were aware of the existence of THN and a minority had THN in their possession. Out of the 34 participants, 29 assessed their own risk of future opioid overdose: 65.5% reported having zero risk, while 6.9% believed they had a high risk. Nevertheless, at least one risk situation of opioid overdose was identified according to HAS criteria in 73.5% of the participants (*n* = 25). Consequently, 55% of the participants underestimated their risk of experiencing a future opioid overdose. Yet, dispensing THN has been judged necessary for 88.2% of the participants.

**Conclusion:**

This study underscored the imperative need to inform not only healthcare professionals but also the patients and users themselves on the availability of THN and the risk situations of opioid overdose.

## Introduction

Opioid-related mortality represents a significant global public health concern. In France, the number of opioid overdose deaths rose by 146% between 2000 and 2015, from 1.3 to 3.2 deaths per million inhabitants [1], and opioids were implicated in 75% of overdose deaths documented by the French national survey DRAMES (Deaths Related to Drug and Substance Abuse, in French) in 2021. While we are far from the figures of the American epidemic, France was identified in a recent disproportionality analysis conducted using the World Health Organization's pharmacovigilance database as one of the six countries exhibiting the highest indicators of opioid drug abuse and dependence. Notably, the USA was also part of this group [2]. For several consecutive years, methadone has been the primary substance involved in France, surpassing both heroin and buprenorphine. In the year 2020, opioid substitution treatment (OST) was associated with 49% of deaths related to substance and drug abuse [3, 4]. In France, buprenorphine and methadone have been approved as OST since 1995. Buprenorphine is recognized as the primary OST, while methadone is more commonly prescribed for refractory cases and/or complex situations, but the initial choice of an OST is also made in consultation with the patient, taking into consideration their preferences, comorbidities, medical history, potential drug interactions with concurrent treatments, safety considerations, including the risk of diversion via alternative routes of administration (intranasal or injectable) [5]. Both methadone and buprenorphine can be obtained at community pharmacies upon presentation of a medical prescription. By calculating the ratio of individuals receiving OST to the estimated number of people with problematic opioid use, France's OST coverage rate is estimated at 87%. This places France in the third position within Europe [6].

Early administration of naloxone, a competitive opioid receptor antagonist, could prevent 4 out of every 5 deaths from opioid overdose [7]. In France, naloxone is available in 3 different forms: (i) an IV form, for hospital use only [8], and 2 "ready-to-use" forms (“take-home naloxone,” THN), intended for outpatient use and usable by all: (ii) a form for intranasal administration, available in pharmacies on prescription and reimbursable [9], and (iii) a form for intramuscular administration, available in pharmacies without prescription and reimbursable upon presentation of a prescription [10]. In France, obtaining THN free of charge in pharmacies currently requires presenting a prescription. This stands in contrast with the noteworthy example of emergency contraception, which has been accessible in pharmacies without a medical prescription or upfront payment for any woman since January 1, 2023 [11]. However, intranasal and intramuscular forms can also be given free of charge to users in specialized addiction treatment facilities.

Although the usefulness of THN and its safety in use are unanimously recognized [12, 13], there are a number of barriers to its distribution among opioid users in France and many other countries. The literature is replete with studies that evaluate these barriers, primarily focusing on healthcare professionals and healthcare systems, with fewer studies delving into the patient perspective. Some of these barriers include time constraints, follow-up challenges, and financial burdens for patients. However, the most frequently cited obstacles are a lack of knowledge about naloxone and difficulties in identifying the appropriate target patients [14–17]. To the best of our knowledge, all these studies have examined these challenges solely from the perspectives of either healthcare professionals or patients, with none offering a comparative analysis of the two viewpoints. The objective of this article was to compare patients' self-assessment of the risk of future opioid overdose with the hetero-assessment provided by healthcare professionals in a population of individuals eligible for naloxone.

## Methods

### General design of the study

This was a multicenter descriptive observational study carried out in pharmacies across the Pays de la Loire region (PDL, France) during April and May 2022. The data for this study were collected by 6 pharmacists, situated in various settings, including rural, urban, and seaside areas across three departments, who had been employed in the clinical pharmacology department of Nantes University Hospital for the preceding 5 years and were currently working in pharmacies, and thus had specific expertise in addiction surveillance. In France, in 2022, the High Health Authority (HAS) issued recommendations concerning the appropriate use of opioid medications, aiming to enhance their safety, increase the awareness of healthcare professionals in identifying and offering early intervention for problematic drug use, and encourage the widespread distribution of THN. Specifically, the HAS recommended that any healthcare professional caring for patients on opioids systematically assesses the appropriateness of prescribing and dispensing naloxone for all opioid users. The HAS identified specific situations as posing a higher risk of overdose, including patients receiving an OST [18]. We have therefore chosen to focus on this population of patients. All adult patients who visited a participating pharmacy for a prescription of buprenorphine or methadone as part of OST and provided oral informed consent were enrolled in the study. Retrospective data were collected through cross-sectional interviews conducted by the pharmacist with the patient, utilizing *an *ad hoc questionnaire. In accordance with current French law, the study was approved by a local ethics committee on March 2022.

### Number of subjects

It is challenging to determine the required number of subjects for this study, as it is exploratory, and there is a lack of existing literature on subjects' self-assessment of overdose risk or pharmacist assessment based on the HAS criteria published in 2022. Consequently, we opted to include pharmacists trained across the region over a specified collection period, rather than attempting to estimate a specific number of subjects.

### Data collection

The following data were collected:

- General characteristics: socio-demographic data of the participants (age and sex), data relating to OSTs (prescribed molecule and history of use; substances at the origin of the initiation of the OST; identification of the OST prescriber) and to THN (patient's knowledge about THN, wish to own THN, knowledge of where THN are available, personal previous use of THN).
- Assessment of the patient's risk of future opioid overdose:
- Patient self-perceived risk of future opioid overdose. Patients were asked to rate their risk using a Likert scale ranging from 0 to 10, where 0 was no risk of future opioid overdose and 10 was extremely high risk of future opioid overdose.
- Pharmacist assessment of the same patient’s risk of future opioid overdose. Overdose risk was assessed using situations identified as being at greater risk of overdose by the HAS: history of OST interruption and/or transition period (criterion 1) (the periods of interruption must correspond to real breaks, such as a minimum stoppage of more than 3 to 5 days, taking into account the half-lives of various OSTs), OST overconsumption (criterion 2), diversion of the OST route of administration (criterion 3), concomitant consumption of a substance having a depressant effect on the central nervous system (criterion 4), patient who had previously used naloxone (criterion 5).
- THN acceptance among patients prescribed OST: history of offering or requesting THN, actual acquisition, and information given to family/friends (if applicable).

*Outcomes*

- Primary outcome: cross-assessment: comparison of the assessment of the risk of opioid overdose between the patient and the pharmacist.
- Secondary outcome: assessment of the need to dispense THN: dispensing THN has been judged necessary when at least one of the following criteria was validated:
- detection of at least one risk situation by the pharmacist
- patient self-assessment of the risk of future overdose > 5 on the Likert scale
- patient wishing to hold THN.

### Data analysis

We carried out a descriptive analysis of all the variables in the analysis: median and interquartile for quantitative variables, numbers, and percentages by modality for qualitative variables.

## Results

### General characteristics

A total of 34 patients were interviewed in the 6 participating pharmacies: Loire Atlantique (65%, *n* = 22), Vendée (29%, *n* = 10) and Sarthe (6%, *n* = 2). Table 1 shows characteristics of the sample.

**Table 1:** Characteristics of the sample

	*n* (%)
Socio-demographic data	
Median age (in years) [interquartile]	39 [35–47]
Sex: male	23 (67.6)
Prescribed OST	
Methadone	21 (61.7)
Buprenorphine	13 (38.3)
Median history of prescription (in years) [interquartile]	8 [5–10]
Substances at the origin of the initiation of the OST	
Heroin	24 (70.6)
Buprenorphine (non-prescribed)	4 (11.8)
Codeine	1 (2.9)
Morphine	1 (2.9)
Other/unknown	4 (11.8)
OST prescriber	
General practitioner	25 (73.5)
Hospital practitioner	4 (11.8)
Treatment Center	5 (14.7)
THN	
Patient’s knowledge about THN (Yes)	11 (32.4)
Desire to own THN (Yes)	18 (52.9)
Knowledge of where THN are available (Yes)	13 (38.2)
Personal previous use of THN (Yes)	0

Participants were mostly men around 40 years old with a long-standing prescription. Methadone was the most prescribed OST, reported in 61.7% of cases. Heroin was the substance at the origin of the initiation of the OST in nearly ¾ of cases. In the majority of cases, the OST was prescribed by a general practitioner (73.5%, *n* = 25).

Of the 34 participants, only 32.4% (*n* = 11) were aware of the existence of THN: 3 through healthcare professionals or professionals working in a specialized addiction treatment facility and 4 through other channels (media, *n* = 3, another user, *n* = 1). The sources for 4 patients were not disclosed. One-third of the participants were aware of the locations to obtain THN, with the majority mentioning pharmacies as the primary source. At the end of the interview, 52.9% (*n* = 18) of participants said they would like to have THN available. Over half of the participants (55.5%, *n* = 10) who expressed a desire for THN were initially unaware of its existence at the beginning of the interview. None of the patients surveyed had prior experience using THN, either for themselves or for assisting someone else.

### Patient self-perceived risk of future opioid overdose

Out of the 34 participants, 29 assessed their own risk of future opioid overdose using a scale from 0 (indicating no risk) to 10 (maximum risk). These participants generally rated themselves as having a low risk of future opioid overdose, with a median rating of 0 out of 10 and an average rating of 1. Approximately 65.5% of patients (*n* = 19) reported having zero risk of experiencing a future opioid overdose. Among those who assigned a non-zero score, 27.6% (*n* = 8) indicated a risk level of less than 5, while 6.9% (*n* = 2) believed they had a high risk, scoring above 5 on the scale.

### Pharmacist assessment of the same patient’s risk of future opioid overdose

Pharmacists identified at least one of the five criteria for assessing the risk of opioid overdose in 73.5% of the participants (*n* = 25). Among the 34 participants, 23.5% (*n* = 8) had one risk factor, 32.3% (*n* = 11) had two risk factors, and 17.7% (*n* = 6) had three risk factors. The most frequently identified risk situations, in order of frequency, were as follows:

(i) Criterion 1, which was present in over half of the patients (58.8%, *n* = 20), involved the following situations: 16 patients reported experiencing interruptions in their OST, while 14 patients reported transitioning periods associated with life changes, such as hospitalization. Notably, 10 patients reported experiencing both periods of interruption and transition.
(ii) Criterion 4, which involves the consumption of substances with a depressant effect on the central nervous system, was reported by 44.1% of patients (*n* = 15). The substances reported in this category were benzodiazepines (*n* = 8), alcohol (*n* = 5), and opioid analgesics (such as codeine, tramadol, and morphine; *n* = 4). Notably, 26.5% of patients (*n* = 9) reported using at least two central nervous system depressants in addition to their OST. Occasional use of heroin was reported in two patients, but not in conjunction with OST—both cases involved methadone. Additionally, three patients reported using cannabis.
(iii) Criterion 2, which was observed in approximately one-third of the patients (35.3%, *n* = 12). These patients reported experiencing episodes of overuse of their OST, with such behavior often occurring during the initial stages of treatment and sometimes driven by a desire for anxiolytic-like psychoactive effects.
(iv) Criterion 3, which pertains to the diversion of the route of administration, was rarely observed, with only one patient reporting that he or she had occasionally taken its OST via nasal administration.

None of the participants reported any prior use of naloxone (criterion 5).

### Cross-assessment

**Fig. 1:**
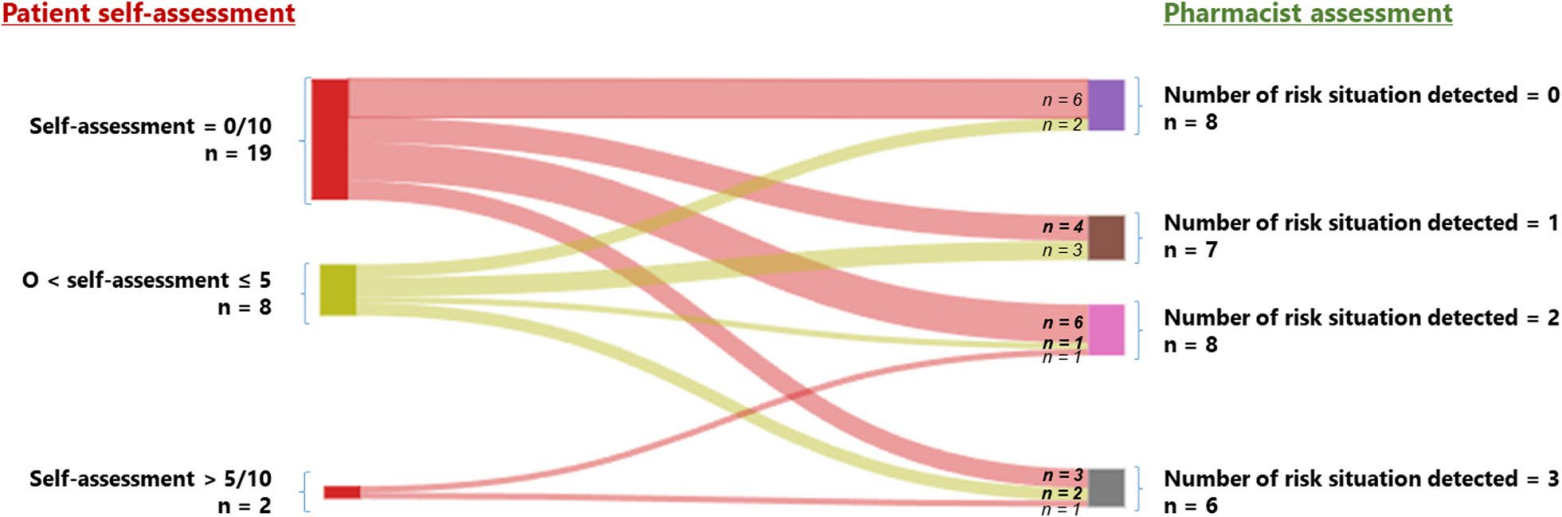
Cross-assessment of the patient's risk of future opioid overdose by the patient and by the pharmacist

Figure 1 displays the agreement in the assessment of the risk of future opioid overdose between the patients and the pharmacists among the 29 participants who self-assessed their risk.

The left scale shows the patient self-assessment as rated in the Likert scale, divided in 3 categories. The right scale indicates the number of risk situation detected by the pharmacist among the same patient.

Out of the 29 patients, only 45% (*n* = 13) had assessments that aligned with the pharmacist's hetero-assessment. This subgroup consisted of: 6 patients in whom the pharmacists did not identify any overdose risk situations, and these patients self-assessed their risk at 0; 5 patients who perceived a low risk, and the pharmacist found 0 to 1 risk factor; 2 patients who self-assessed a high risk and indeed had at least 2 risk factors detected by the pharmacists.

Conversely, 55% (*n* = 16) of participants underestimated their risk of future opioid overdose. Notably, among the 19 patients who self-assessed a zero risk, 68% of them (*n* = 16) had at least one risk factor for overdose as identified by the pharmacist and up to 3 risk factors for 3 of them (15.8%).

### Assessment of the need to dispense THN and THN acceptance among the participants

Among the 34 participants in this study, the need to dispense THN has been judged necessary for 30 patients (88.2%). These patients validated at least one of the following criteria:

- Detection of at least one risk situation by the pharmacist: **25 patients** were identified.
- Patient self-assessment of the risk of future overdose > 5 on the Likert scale: 2 patients were identified. For each of them, the pharmacist detected at least one risk situation of overdose.
- Patient wishing to hold THN: 18 patients were identified. Table 2 displays the distribution of participants by the number of risk situations detected by the pharmacist and their respective desire to possess THN. The wish to hold THN, which was expressed without knowing the results of the cross-evaluation, was the highest (64.7%, *n* = 11) among the 17 patients who presented at least two criteria for assessing the risk of opioid overdose; three of them were already in possession of THN at the time of the interview. Notably, there were **5 patients** who expressed a desire for THN, despite the pharmacist not detecting any risk situation of overdose in their assessment.

**Table 2:** Need to dispense THN among the participants and corresponding wish to possess THN

Number of risk situation detected by the pharmacist	Number of participants concerned (*n* = 34)	Number of participants wishing THN *n* (% of participants concerned)
0	9	5 (55.5)
1	8	2 (25.0)
2	11	6 (54.5)
3	6	5 (83.3)

Among the 34 participants, five patients had either been offered THN or had requested it from a healthcare professional prior to their participation in this study. All of them actually owned THN, and four of them had informed their family and friends about it. Therefore, the acceptance of THN among these patients was notably high, standing at 80%.

## Discussion

### THN: low distribution in a high-risk population…

Out of the 34 patients receiving OST in our study, only 14.7% (*n* = 5) had access to THN. This proportion is in line with national estimates. In 2019, it was estimated that 177,000 individuals were prescribed OST in France. During the same year, 7667 THN kits were ordered in France by pharmacies, hospitals, and specialized facilities, and these kits were not exclusively provided to patients on OST [6]. Based on these figures, it appears that less than 23% of patients on OST were likely to have access to THN. However, the HAS identifies patients on OSTs, especially those on methadone, as one of the populations at highest risk of overdose, especially during the initiation and discontinuation of treatment [18]. This risk factor is found in several international studies and official recommendations [19–21]. Indeed, there are two primary periods of elevated risk of mortality for patients on OSTs: the initiation of treatment and the month following treatment cessation. These increased risks are largely attributed to the decreased tolerance to opioids during these transitional periods [22, 23]. According to the College of Psychiatrists in Ireland, the risk of a fatal overdose within 15 days of initiating methadone treatment is estimated to be 6–7 times higher than in heroin users who are not receiving OST and a striking 98 times higher than in patients who are well stabilized on OST [24]. According to Webster et al., death occurs within 7 days of the start of methadone treatment in 70% of methadone-prescribed overdose victims [22]. Finally, according to a systematic review of the literature, the first 4 weeks following initiation of methadone treatment and following cessation of treatment are the periods most at risk of overdose: 11.4 deaths/1000 users and 32.1 deaths/1000 users, respectively [23]. In our study, although 100% of the patients had this risk factor since it was an inclusion criterion, 65.5% of the patients claimed that they had no risk of experiencing a future opioid overdose.

### … despite being in favor of its detention

In our study, all participants who had been offered THN or had requested it from a healthcare professional before their participation actually possessed THN, and 80% of them had informed their family and friends about it. While we acknowledge that our sample size is relatively small and might have some recruitment bias, it does align with a broader trend observed in the literature. For instance, in the French SINFONI study, none of the 355 healthcare professionals mentioned patient refusal as a reason for not prescribing or dispensing THN. Similarly, among the barriers to dispensing naloxone, patient refusal was reported in only 11.6% of cases by opioid treatment program staff [17]. This suggests that patient acceptance of THN may be higher than expected, and the main challenges in expanding its availability might be related to other factors. In our study, the alignment between the need to hold THN and the actual possession of THN was found to be inadequate. Out of the 30 patients for whom the dispensing of THN was considered necessary, only 13.3% (*n* = 4) had THN in their possession. This indicates that a significant portion of patients who are eligible to have THN do not currently possess it. Why is this?." 

### The main patient-related barriers: a lack of knowledge about the existence of THN... 

Out of the 34 participants, only 32.4% (*n* = 11) were aware of the existence of THN. The rate of awareness about naloxone varies in the international literature and may be influenced by selection bias related to the specific population under study. For instance, in a study conducted at a residential substance use treatment center, a notably high 95% of the sample was able to correctly identify naloxone as the "overdose drug"[25]. However, this lack of consumer awareness of the existence and usefulness of THN is widely reported in the literature as one of the main difficulties in distributing THN [17, 26–28]. It is also worth highlighting in our study that, by the end of the interview, more than half of the 18 participants who expressed a desire for THN were initially unaware of its existence. This suggests that enhancing communication about the existence and significance of THN to all opioid users is a primary strategy to promote the wider dissemination and adoption of THN.

### … and an underestimation of the risk of overdose

In addition to the inherent risks associated with the use of OST, our study reveals that patients are largely unaware of the risk of overdose situations. As much as 55% of the participants underestimated their risk of experiencing a future opioid overdose. This challenge has been recognized in several studies involving patients on OST, which have also identified that a lack of communication on the part of the prescriber and pharmacist contributes to reinforcing self-perceptions of a low risk of overdose [25, 28]. The underestimation of the risk of overdose has also been observed in a study involving incarcerated opioid users: participants demonstrated a strong awareness of the increased risk of overdose following their release from prison. However, they displayed little inclination to purchase THN from pharmacies after their release, primarily due to a perception of having a low personal risk of overdose [29]. Finally, one of the obstacles identified in a study of illicit drug users was a feeling of "product control" on the part of users [30].

The most commonly reported risk criterion among participants in our study was a history of OST interruption and/or transition periods, which was present in 58.8% (*n* = 20) of the patients. A loss of tolerance to opioids can occur in as little as 3 days without use. Many publications have identified loss-of-tolerance situations as particularly high-risk of opioid overdose [20, 20, 31–37]. For instance, in Ireland, official recommendations specify that OSTs should not be provided to patients who have experienced a treatment interruption of 3 days or more, unless the prescriber confirms the appropriate dose to be dispensed [24]. Among these situations, the periods of incarceration and release from incarceration are the best described [38]. A similar phenomenon exists for patients leaving a care center [19, 24, 33, 36, 39].

The second most frequently reported risk situation, found in 44.1% (*n* = 15) of participants, was the consumption of a substance having a depressant effect on the central nervous system (CNS): benzodiazepines (*n* = 8) and alcohol (*n* = 5) in the forefront. The summary of product characteristics for methadone and buprenorphine mention an increased risk of sedation, respiratory depression, coma, and death in the event of concomitant use of sedative substances. A large number of publications warn that the concomitant use of CNS depressants increases the risk of opioid overdose [20, 32, 36, 37, 40]. Alcohol is the substance most commonly found with opiates in overdose victims [22]; its use significantly increases the risk of overdose [41]. Concomitant use of benzodizepines is found in 30% of cases of fatal overdose [42, 43], and, according to Sun et al., opiate users taking benzodiazepines are 2.14 times more likely to be admitted to hospital for opiate overdose than opiate users not taking benzodiazepines [44]. However, the way in which the benzodiazepine is obtained and its dose consumed seem to be factors to be taken into account when assessing the risk [21]. Finally, it should be noted that in a study carried out in patients on OSTs, methadone taken in combination with alcohol or benzodiazepines was perceived at low risk by 55% of patients [45].

Enabling patients to have a more accurate assessment of their individual risk of overdose could indeed be a second effective strategy. It is worth noting that in our study, among the patients at the highest risk of overdose (comprising 50.0% of all participants), two-thirds expressed a desire to possess THN by the end of their interview. These high-risk situations can be identified by prescribing doctors and/or pharmacists during the dispensing of OST. A systematic evaluation of these factors by healthcare professionals could empower both professionals and users to more effectively assess the risk of overdose and the importance of having THN on hand.

### Need for shared medical decision-making

Some risks associated with overdose, particularly periods of tolerance loss, are well documented, as mentioned earlier. However, we demonstrate that very few patients receive naloxone despite their willingness to have it when questioned, even though they often underestimate their risk of overdose. Why does this occur? The first hypothesis is the insufficient awareness of opioid overdose prevention methods, both among healthcare professionals and patients. This lack of awareness may result in the prescription and guidance for THN not being an automatic consideration for healthcare professionals. The limited awareness regarding naloxone may explain the low risk assessment by healthcare professionals and patients, particularly given that official recommendations outlining at-risk situations were only clearly defined in France as recently as 2022. The pharmacists participating in this study were selected because they were trained in these recent recommendations, given their experience in pharmacology. The second explanation could be the difference in the dispensing modalities (over-the-counter or prescription) in France, which may create a sense of ambiguity and potentially associate certain forms with risks, even though it is not the case. Finally, the need for a prescription to receive reimbursement in an economically disadvantaged population may also explain why pharmacists are hesitant to assess overdose risks and dispense without a medical prescription. It seems urgent to implement training actions for healthcare professionals, as well as information campaigns for users to raise awareness about the importance of having naloxone to reduce the risks of death in case of overdoses. In the era of shared medical decision-making, it is also crucial to encourage shared risk assessments between users and healthcare professionals to improve the availability and acceptability of naloxone.

### Strengths and limitations

This study marked a pioneering endeavor in objectively assessing the risk of overdose, as perceived by healthcare professionals and patients alike. Moreover, it afforded us the opportunity to delineate the predominant obstacles on the patient side hindering the distribution of THN to an eligible population, a significant portion of whom exhibited multiple risk factors for opioid overdose. Furthermore, it facilitated the evaluation of the imperative need to dispense THN to patients undergoing OST at pharmacies, as well as the acceptance of THN among these individuals. It is worth noting that the participant count in this study is relatively modest, due to the small number of pharmacies that met the criteria for taking part. It is therefore not necessarily in alignment with the prevailing landscape of OST utilization in France. In 2021, nearly 162,000 individuals acquired OSTs from French pharmacies, with the majority of these prescriptions originating from general practitioners.

We cannot be sure that our sample is representative of patients receiving OST, either in terms of socio-demographic characteristics or treatments received.

Moreover, buprenorphine emerged as the most frequently prescribed OST, accounting for 55% of patients. Nevertheless, the proportion of beneficiaries receiving methadone prescriptions continues to exhibit an upward trajectory (38% in 2017 vs. 44% in 2021) [6]. In our study, it is notable that the percentage of participants using methadone (61.7%) appears to surpass the proportion observed among all OST users in France. This could be related to geographical characteristics but also to the fact that the participating pharmacists could have considered methadone patients to be at a higher risk of overdose, leading to a higher likelihood of their inclusion in the study. This perception aligns with the fact that methadone is the substance most frequently implicated in overdose deaths, significantly exceeding the involvement of buprenorphine.

Nevertheless, our study is exploratory in nature and focuses on analyzing predominantly qualitative data. Our goal is not to achieve representativeness but rather to underscore the divergence in assessments between users and healthcare professionals. For healthcare professionals, a thorough evaluation of the necessity for prescribing naloxone and its acceptability to the patient should be conducted systematically. This evaluation should lead to a collaborative medical decision, acknowledging potential disparities between the two perspectives.

It is noteworthy to mention that within the scope of our study, none of the participants had prior experience with THN, either for their own use or for assisting others. Numerous research endeavors have demonstrated a substantial correlation between a prior overdose event, whether experienced personally or observed as a bystander, and an elevated perception of heightened overdose risk [46] and a facilitator for holding THN [47], whereas no prior traumatic experience of overdose in patients on OST is a barrier [25, 28]. Nonetheless, several studies have documented a hesitancy to utilize THN among patients who have previously endured withdrawal symptoms after its administration in the context of a prior overdose [48–50]. Furthermore, the methodology employed in our study may have obscured an additional barrier that has been highlighted in the existing literature: the apprehension of stigma among individuals grappling with addiction, which could lead them to be hesitant in disclosing their addiction issues to pharmacy personnel. Moreover, there exists a concern regarding the potential negative response from patients toward healthcare professionals (48). In our study, the incorporation of both healthcare professionals and patients necessitated their consent, which inherently assumed an absence of hesitancy in discussing the topic. Each patient was appraised in advance that their responses to the questionnaire, as well as their decision to participate or decline, would have no bearing on their customary medical care.

## Conclusion

This study underscored the imperative to broaden the scope of training and information dissemination pertaining to the availability of THN and the risk situations of opioid overdose, reaching not only healthcare professionals but also the patients and users themselves. A substantial segment of patients and users remains unaware that opioid overdoses result from a complex interplay of various factors, necessitating comprehensive consideration when prescribing and dispensing opioids. This holistic evaluation is essential for accurately assessing an individual's risk of opioid overdose and the suitability of providing them with THN.

## Data Availability

The datasets used and/or analyzed during the current study are available from the corresponding author on reasonable request.
